# Plant-Derived Treatments for IBS: Clinical Outcomes, Mechanistic Insights, and Their Position in International Guidelines

**DOI:** 10.3390/nu18020183

**Published:** 2026-01-06

**Authors:** Ploutarchos Pastras, Ioanna Aggeletopoulou, Maria Bali, Christos Triantos

**Affiliations:** Division of Gastroenterology, Department of Internal Medicine, University of Patras, 26504 Patras, Greece; ploutarchosp96@gmail.com (P.P.); mariabalimed@gmail.com (M.B.); chtriantos@upatras.gr (C.T.)

**Keywords:** Irritable Bowel Syndrome, plant extracts, herbal medicine, treatment, mechanisms of action, peppermint oil, international guidelines

## Abstract

Irritable Bowel Syndrome (IBS) affects 4–15% of the global population, and the limited efficacy of existing pharmacologic therapies has driven growing interest in plant-based therapeutic options among both patients and clinicians. A comprehensive assessment of all plant extracts investigated in IBS is therefore essential, given the limited effectiveness of conventional treatments and the increasing interest in complementary approaches. Evidence from recent systematic reviews and meta-analyses consistently indicates that peppermint oil is the most effective botanical agent, particularly for reducing abdominal pain and overall IBS symptom severity. Iberogast (STW-5 and STW-5 II) has also demonstrated clinical improvements across multiple trials, while curcumin shows mechanistic and preliminary clinical potential by modulating several IBS-related pathways. In contrast, extracts such as *Curcuma xanthorrhiza*, *Fumaria officinalis*, and various Ayurvedic formulations have not shown significant clinical benefit. Other agents, including *Aloe vera*, flavonoids, St John’s wort, and ginger, exhibit mixed or inconsistent results, reflecting heterogeneity in study designs and underlying mechanisms. A review of international guidelines reveals that peppermint oil is the only plant-based therapy consistently acknowledged across adult and pediatric recommendations. The aim of this review is to summarize, compare, and critically evaluate all plant extracts studied for the prevention and treatment of IBS, integrating mechanistic pathways, clinical evidence, and current international guideline recommendations to clarify their therapeutic relevance for clinical practice.

## 1. Introduction

Disorders of gut–brain interaction (DGBIs) are functional gastrointestinal conditions characterized by a complex interplay between the gut and the brain [1]. A recent epidemiological study estimates that the global prevalence is approximately 40%, with rates increasing following the COVID-19 pandemic [2]. One of the most widely investigated DGBIs is Irritable Bowel Syndrome (IBS), which affects between 4% and 15% of the population worldwide, depending on geographic region and diagnostic criteria [1,3]. Young adults, particularly females, have a higher prevalence, which has increased substantially over the past decades [1]. The etiology of this condition remains unclear across many aspects of its pathophysiology. Various mechanisms are known to be involved, including the interaction of the microbiota–brain–gut axis, immune activation, the hypothalamic–pituitary–adrenal axis, visceral hypersensitivity, and environmental factors such as psychological distress and diet [4]. Additionally, the enteric nervous system (ENS) and the gut microbiome play a significant role, mainly in regulating intestinal motility [5].

Due to this multifactorial and unclear background, the most recent diagnostic criteria (Rome IV) are based on clinical symptoms [6]. More specifically, the presence of recurrent abdominal pain, on average, at least 1 day per week in the last 3 months, associated with two or more of the following: symptoms related to defecation, a change in stool frequency, or a change in stool form [6]. Furthermore, IBS is divided into four subtypes: predominantly loose and frequent stools [IBS-Diarrhea (IBS-D)], infrequent and hard stools [IBS-Constipation (IBS-C)], alternating diarrhea and constipation [IBS-Mixed (IBS-M)], and patients who meet the Rome IV criteria but whose bowel habits cannot be reliably classified into one of the other groups [IBS-Unclassified (IBS-U)] [7].

Although IBS is a functional disorder without an adverse prognosis for life expectancy, it negatively affects quality of life. IBS is associated with reduced engagement in daily activities, increased work absenteeism, and impaired functioning, thereby increasing the global healthcare burden [8]. To address this disorder, many therapies have been developed, with varying and sometimes controversial results [9]. The multitude of underlying IBS mechanisms contributes to a heterogeneous set of treatment options that may relieve symptoms in different patient groups [10]. Pharmacological therapy is used to manage predominant symptoms, such as constipation or diarrhea; however, these treatments are unsatisfactory for many patients, because they do not modify the long-term course of the disease, typically target only a single underlying etiological mechanism, and are often associated with side effects [11]. Therefore, dietary, complementary, and alternative medicines (CAMs) gain increasing interest in IBS management [11,12]. The most commonly used CAMs include relaxation techniques, nutritional supplements, and herbal medicines [13]. More specifically, plant extracts are gaining ground, as patients increasingly turn to them when conventional treatments fail to resolve symptoms and because they target multiple underlying mechanisms [14]. The two major categories of herbal medicines in IBS are Traditional Chinese Medicine (TCM) [15,16], one of the world’s major herbal medicine traditions, and Western Herbal Medicine (WHM), which has European origins with influences from North America and Australia [12]. Numerous studies have assessed the efficacy of plant extracts in IBS, with findings varying from beneficial to ineffective and, in some cases, inconclusive.

This narrative review aims to summarize and compare the plant extracts studied for IBS prevention and management, providing clinicians and patients with a clear overview of their potential role. More specifically, the review seeks to critically evaluate all available plant-derived interventions by integrating mechanistic pathways, clinical evidence, and current international guideline recommendations, thereby clarifying their therapeutic relevance and helping inform evidence-based clinical practice.

## 2. Effective Plant Extracts in Irritable Bowel Syndrome Management

### 2.1. Peppermint Oil

Mint has been used worldwide for decades to relieve gastrointestinal disturbances due to its soothing effects. Peppermint is a common perennial herb in North America and Europe, and nowadays, peppermint oil, as a WHM therapy, is widely used in DGBI treatment and is the most studied plant extract in IBS [17]. Peppermint oil is extracted from the perennial flowering plant *Mentha piperita* and contains menthol (30–35%), menthone (14–32%), limonene (1–3.5%), 1,8-Cineole (3.5–8%), menthofuran (1–8%), isomenthone (1.5–10%), menthyl acetate (2.8–10%), pulegone (<3%), carvone (<1%), isopulegol (<0.2%). Menthol is the primary bioactive component [18]. The commercial preparation shown to improve abdominal pain and other gastrointestinal (GI) symptoms in patients with IBS is enteric-coated peppermint oil, which prevents its release in the esophagus or stomach [19].

The effect of peppermint oil in IBS results from several pathophysiological pathways. Menthol enhances inhibitory smooth muscle contraction while simultaneously stimulating GI motility [20,21]. More specifically, it blocks L-type calcium channels in gastrointestinal smooth muscle, thereby inhibiting smooth muscle contractions and relieving colonic spasms. In contrast, menthol increases calcium influx in mouse dorsal root ganglionic neurons, colonic organoids, and peritoneal macrophages through activation of transient receptor potential cation channel, subfamily A, member 1 (TRPA1) channels [22,23]. In addition, menthol modulates TRPA1 activation in interstitial cells of Cajal (ICCs), the pacemaker cells responsible for slow waves in GI smooth muscle, thereby depolarizing the membrane potential in the murine small intestine and stimulating GI motility [21]. Peppermint oil also modulates the gut microbiome. Microbiota composition is affected by the antimicrobial, anti-inflammatory, and antifungal properties of menthol, which are mediated by its modulation of pro-inflammatory cytokine levels and immune system function [24]. Furthermore, peppermint oil decreases the Firmicutes/Bacteroidetes ratio, thereby altering the stool microbiome in children with functional abdominal pain disorders, a change that correlates with improvement in IBS-like symptoms [25]. Beyond its effects on GI motility and the microbiome, menthol influences other pathophysiological mechanisms associated with IBS, such as analgesic effects via inhibition of 5-HT3 serotonin and gamma-aminobutyric acid (GABA) receptors, potassium and voltage-gated sodium channels, and transient receptor potential cation channels in vitro [26,27].

The above-mentioned pathophysiological mechanisms of peppermint oil in alleviating IBS symptoms have been supported by multiple studies, demonstrating its efficacy as a promising therapeutic option for this disorder [28]. Clinical trials conducted before the Rome IV criteria showed that a 4-week treatment with peppermint oil (two capsules twice daily) improved IBS abdominal symptoms compared with placebo (*p* < 0.009) in a prospective double blind randomized controlled trial [29], and an 8-week treatment (one capsule three times daily) reduced abdominal pain or discomfort compared with placebo (*p* < 0.001) in a randomized double-blind placebo-controlled study with 90 participants [30]. Consistent with the Rome IV criteria, a randomized, double-blind trial of 190 patients with IBS in the Netherlands showed that small-intestinal-release peppermint oil (182 mg three times daily) resulted in greater improvements in secondary outcomes, such as abdominal pain (*p* = 0.016), abdominal discomfort (*p* = 0.020), and IBS symptom severity (*p* = 0.020) compared to placebo, while no significant difference was observed in overall symptom relief (*p* = 0.170) [31]. A recent phase 3 trial observed statistically significant improvements in all IBS symptom severity scores, stool frequency score [at IBS-C *p* = 0.020 (CI: 0.04–0.37) in week 2 and *p* = 0.026 (95% CI: 0.03–0.32) in week 4], patient’s global assessment (PtGA) [71.6% (95% CI: 59.4–81.9) in week 2, 85.1% (95% CI: 74.3–92.6) in week 4], and stool form score [at IBS-D *p* = 0.001 (95% CI: −0.61–−0.16) in week 2] in 69 Japanese patients (one capsule three times daily before meals), confirming findings from previous clinical studies in Western countries [32]. Regarding adverse events, no serious events were reported; those observed were mild and transient, including heartburn [29,31,32], belching with minty taste [31], peppermint-oil-scented stool [31] and hypersensitivity/eczema [32]. Only the most recent phase 3 trial provided explicit subtype-stratified improvement rates, with the highest Patient Global Assessment (PtGA) improvement observed in patients with IBS-M; however, the study design and small subtype-specific sample sizes limit definitive conclusions [32]. Across the above studies, sex-stratified efficacy results were not reported [29,30,31,32]. The efficacy of peppermint oil in IBS is also corroborated by two recent systematic reviews and meta-analyses of eligible trials, which found that this plant extract was safe and superior to placebo in reducing abdominal pain and global IBS symptoms. However, to date, no well-designed head-to-head studies comparing peppermint oil with commonly used IBS medications have been published, nor are there methodologically robust studies evaluating the parallel use of peppermint oil with standard pharmacological treatments [33,34]. A non-inferiority, double-blind randomized controlled trial comparing enteric-coated peppermint oil with a standard antispasmodic agent (e.g., mebeverine) in adult IBS patients meeting Rome IV criteria is currently ongoing, but results have not yet been published (NCT06914921).

### 2.2. Iberogast (STW 5 and STW 5-II)

STW 5 and STW 5-II are herbal combinations widely used in Western countries as part of WHM. STW 5 (Iberogast Classic) developed in Germany in 1960, is a blend of nine hydroalcoholic (31% alcohol) herbal extracts: chamomile flower *(Matricariae flos* from *Matricaria chamomilla*; 20%), bitter candy tuft (*Iberis amara*; 15%), lemon balm leaf (*Melissae folium* from *Melissa officinalis*; 10%), caraway fruit (*Carvi fructus* from *Carum carvi*; 10%), liquorice root (*Liquiritiae radix* from *Glycyrrhiza glabra*; 10%), peppermint leaf (*Menthae piperitae* folium from *Mentha x piperita*; 5%), angelica root (*Angelicae radix* from Angelica archangelica; 10%), milk thistle (Cardui mariae fructus from *Silybum marianum*; 10%), and greater celandine herb (*Chelidonium majus*; 10%) [35]. STW 5-II (Iberogast ADVANCE) was subsequently developed to target the stomach and colon. Compared with STW 5, STW 5-II omits angelica root, milk thistle, and *Chelidonium majus*, and increases the proportions of *Matricariae flos* to 30%, *Melissae folium* to 15%, and *Menthae piperitae folium* to 10% [35].

Iberogast exerts its effects in IBS through multiple pathophysiological mechanisms; however, no single compound within the multi-extract mixtures (STW 5 and STW 5-II) has been proven to be more active than the others in IBS. Chamomile, lemon balm leaf, and peppermint leaf block voltage-gated calcium channels, thereby interfering with intracellular Ca^2+^ transport, relaxing gastrointestinal smooth muscles, and exerting a spasmolytic effect [36,37]. STW 5 and STW 5-II also reduce visceral hypersensitivity by inhibiting activation of the dorsal root ganglion (DRGs) neurons [38]. More specifically, STW 5 desensitizes DRG sensory afferents (TRPA1/TRPV1) in the small intestine. At the same time, STW 5-II reduces neuronal activation (ERK1/2 phosphorylation) in the DRGs, thereby decreasing intestinal afferent sensitivity and reversing stress- and post-inflammatory-induced colonic hypersensitivity [39]. These mechanisms help reduce abdominal pain and bloating [40].

In addition, Iberogast exhibits anti-inflammatory properties by inhibiting the expression of inflammatory mediators, including STAT1, NF-κΒ, and iNOS, and by counteracting cytokine-mediated downregulation of the tight junction protein Zonula Occludens-1 (ZO-1) in vitro [41]. Alterations in the gut microbiome further enhance its anti-inflammatory effects. STW 5-II increases the relative abundances of Gram-positive anaerobes (Bifidobacteriaceae, Eggerthellaceae Lachnospiraceae, Ruminocococcaceae, Clostridiales Family XIII, and Erysipelotrichaceae) and marginally decreases the Gram-negative facultative anaerobe Enterobacteriaceae. These shifts improved Caco-2 cell transepithelial electrical resistance, reduced LPS-induced NF-κB signaling, and increased IL-10 production in THP1-derived macrophages [42].

Clinical studies have verified Iberogast’s beneficial effects in IBS. A recent randomized, double-blind, placebo-controlled clinical study in 10 IBS patients with bloating showed that a 2-week treatment with STW 5 (20 drops three times daily) improved colonic gas tolerance (*p* = 0.035) during a gas challenge test and reduced gas-related abdominal symptoms (*p* = 0.016) [43]. A prospective observational study assessing STW 5 (10–20 drops three times daily for one week) in 418 pediatric IBS patients demonstrated improvement in gastrointestinal symptoms score (GIS) (*p* < 0.0001) and good tolerability (only 0.7% of children reported adverse events) [44]. The largest clinical trial of Iberogast in IBS was a multicenter, parallel-group, double-blind, double-dummy, active-controlled randomized clinical trial from 2004, in which adults were randomized to STW 5 (n = 51), STW 5-II (n = 52), or placebo (n = 52). Both STW 5 and STW 5-II (20 drops three times daily for four weeks) modestly reduced total abdominal pain scores (STW 5, *p* = 0.0009 and STW 5-II, *p* = 0.0005) and IBS symptom scores (STW 5, *p* = 0.001; STW 5-II, *p* = 0.0003) after 4 weeks compared with placebo [45].

### 2.3. Curcuma longa or Turmeric (Curcumin)

In addition to its antioxidant and anti-inflammatory effects, *Curcuma longa* (curcumin, demethoxycurcumin, bisdemethoxycurcumin, and sesquiterpenes), and more specifically its active constituent, curcumin, appears beneficial for improving gastrointestinal symptoms in patients with IBS [46]. The main reason may be that curcumin modulates 5-hydroxytryptamine (5-HT) levels, decreasing 5-HT in the colon of IBS rat models and increasing its levels in the hippocampus [47]. 5-HT plays a significant role in dysregulating the gut–brain axis, a major risk factor for IBS development [48]. Furthermore, curcumin improves intestinal motility, alters gut microbiota composition, and increases the serum levels of substance P and vasoactive intestinal peptide in an IBS-C rat model [46].

Clinical studies in humans have shown that turmeric alone (οne or two tablets of 72 mg once daily for 8 weeks) [49] and in combination with Boswellia serrata (17–23% *w*/*w* curcumin in one tablet of 500 mg twice daily for one month) [50] can improve gastrointestinal symptoms in IBS. A recent systematic review and meta-analysis of randomized controlled trials and comparative observational studies, including 551 adults with IBS who received turmeric, reported improvements in overall quality of life and reductions in IBS-SSS scores and abdominal pain; however, many included studies had methodological limitations [51].

### 2.4. Traditional Chinese Medicine (TCM)

Chinese herbal medicine plays a critical role as a potential complementary therapy for IBS. TCM has not yet been studied in Western populations. Three Chinese herbal preparations that have been studied in clinical trials in IBS patients and demonstrated beneficial effects are the following:

#### 2.4.1. JCM-16021

This Chinese herbal formula consists of seven herbs [*Rhizoma Atractylodis Macrocephalae* (Baizhu), *Radix Paeoniae Lactiflorae* (Baishao), *Cortex Magnoliae Officinalis* (Houpo), *Semen coicis Lachryma-jobi* (Yiyiren), Polygonaceae (Huotanmu), *Fructus Terminaliae Chebulae* (Hezi), and *Rhizoma Corydalis Yanhusuo* (Yanhusuo)], with the main classes of active compounds including terpenoids, polysaccharides, glycosides, and polyphenols [52]. JCM-16021 modifies the synthesis and metabolism of 5-HT in the colon of neonatal maternal separation (NMS) rats, attenuating visceral hyperalgesia [53]. In addition, it reduces colonic enterochromaffin (EC) cell hyperplasia and serotonin availability and upregulates mucosal cytokines, particularly T-helper 1 (Th1)-related cytokines, in post-inflammation IBS (PI-IBS) rats [53]. Furthermore, by modulating short-chain fatty acid (SCFA) producers, JCM-16021 relieves abdominal pain in rats, as demonstrated through a fecal microbiota transplantation (FMT) study [54]. Two multicenter, double-blind, placebo-controlled RCTs of 392 participants (1:1 allocation, 196 per arm) evaluated JCM-16021 granules, which contain Baizhu (20 g), Baishao (15 g), Houpo (10 g), Yiyiren (20 g), Huotanmu (20 g), Hezi (10 g), and Yanhusuo (15 g). The formulation was administered orally at a dose of 8 g per sachet, three sachets daily for 8 weeks, 30 min after meals. These randomized clinical trials demonstrated that JCM-16021 alleviates abdominal pain in patients with IBS-D [52,54].

#### 2.4.2. Tong-Xie-Yao-Fang

Tong-Xie-Yao-Fang is the most extensively studied Chinese herbal preparation for IBS and comprises four herbal components: Radix Paeoniae Alba (Baishao), Pericarpium Citri Reticulatae (Chenpi), *Radix Saposhnikoviae* (Fangfeng), and *Rhizoma Atractylodis Macrocephalae* (Baizhu). The main classes of active compounds include monoterpene glycosides, flavonoids, lactones, and chromones. A double-blind, placebo-controlled, randomized trial involving 160 adults reported that this formula (Baishao, Baizhu, Chenpi and Fangfeng) (15 g three times daily for 4 weeks) was more effective than placebo in relieving symptoms in patients with IBS-D [55]. Tong-Xie-Yao-Fang reduces mast cell-mediated visceral hypersensitivity, inhibits colonic smooth muscle hypercontractility, modulates brain–gut axis mediators (e.g., BDNF, serotonin, corticotropin-releasing factor), and restores a physiological gut microbiota, thereby decreasing mucosal serotonin levels [56].

#### 2.4.3. Xiang-Sha-Liu-Jun-Zi Tang (XSLJZT)

XSLJZT, also known as Rikkunshito in Japanese Kampo medicine, is another herbal formula used in both traditional Chinese and Japanese medicine. This formulation contains eight herbal ingredients: *Atractylodes japonica* (Okera rhizome), *Citrus unshiu* (Japanese mandarin peel), *Glycyrrhiza uralensis* (licorice root), *Panax ginseng* (ginger root), *Pinellia ternata* (Pinellia tuber), *Poria cocos* (Poria mushroom), *Zingiber officinale* (ginseng), and *Ziziphus jujuba* (jujube). The main classes of bioactive compounds include nucleosides, lactones, flavonoids, and triterpene acids [57]. Although most of Rikkunshito’s pathophysiological actions have been described in functional dyspepsia (FD), a double-blind placebo-controlled RCT with 80 IBS patients (1:1 40 per arm) showed beneficial effects of XSLJZT (chemical compound as above) (3 g three times per day for 28 days) in alleviating GI symptoms and improving quality of life in patients with IBS-D [58,59].

### 2.5. Kampo (Japanese Traditional Medicine)

Kampo is a system of Japanese phytotherapy with numerous formulations, each produced with standardized proportions of specific herbal ingredients. The Kampo preparations most commonly used in IBS are Keishi-ka-shakuyaku-to, Saiko-keishi-to, Hange-shashin-to, Daikenchuto, and anthraquinone-containing Kampo formulas [60]. These kampo products may influence IBS pathophysiology by modulating gut motility and the gut microbiome, decreasing visceral hypersensitivity, reducing low-grade inflammation, and influencing the gut–brain axis [61]. Kampo therapies are recommended for patients with IBS by the Japanese Society of Gastroenterology and the Second Asian Consensus on IBS [62,63]. Kampo has not yet been studied in Western populations.

### 2.6. Preclinical Evidence for Plant-Derived Compounds in Irritable Bowel Syndrome

Several herbal products have demonstrated beneficial effects in preclinical studies of IBS, although no clinical studies in humans are currently available. In a recent in vitro study, *Scutellaria baicalensis* (baicalin, baicalein, wogonin, wogonoside, oroxylin A) showed anti-inflammatory and analgesic effects in animal models by reducing inflammatory responses and ion channel activity [64]. A recent multi-omic survey showed that *Linderae Radix* (isoquinoline alkaloids, sesquiterpenes/volatiles, flavonoids) ameliorates diarrhea, visceral hypersensitivity, and inflammation in IBS-D animal models by restoring gut microbiota diversity and correcting dysregulated biomarker metabolism [65]. *Serpylli herba* extract (carvacrol and/or thymol, γ-terpinene, *p*-cymene) exhibited anti-inflammatory and visceral analgesic effects in an experimental rat model of IBS by improving gut hypersensitivity, visceral hyperalgesia, and inflammation, potentially through inhibition of mast cell degranulation and modulation of the serotonin pathway [66]. Mao Jian Green Tea ethanol extract (catechins, caffeine, other polyphenols) reduces GI symptoms in a rat IBS-C model by regulating the serotonin pathway and enhancing gut microbiota diversity [67]. Banhasim-tang, a traditional herbal formula, may alleviate IBS-D symptoms by modulating ion channels [68]. Epigallocatechin-3-gallate (EGCG) (C_22_H_18_O_11_), the primary active component of green tea, may help prevent and treat IBS by targeting disease-related mechanisms identified through molecular docking analyses [58].

## 3. Plant Extracts with Controversial Results

### 3.1. Soy and Propolis Flavonoids

Soy (genistein, daidzein, glycitein, and glycoside forms) and propolis (pinocembrin, chrysin, galangin, caffeic acid phenethyl ester) flavonoids are naturally occurring polyphenolic compounds with antioxidant, anti-inflammatory, and antispasmodic properties. They are widely present in fruits and vegetables (e.g., quercetin), various herbs and plants, and soy (the most-studied dietary source in IBS research) [69]. Preclinical studies suggest potential benefits in IBS, but clinical evidence remains inconsistent and limited. Their effects vary by IBS subtype and by the specific type of flavonoid examined [70]. In rat models, flavonoids have been shown to modulate proinflammatory cytokine release, improve gut motility, and attenuate visceral hypersensitivity, exerting a smooth muscle-relaxing effect [71].

A randomized, double-blind clinical trial involving 67 IBS patients showed that soy flavonoids reduced only the IBS-Quality of Life (IBS-QoL) score (*p* = 0.009), but not the IBS-SSS score (*p* = 0.068), compared to placebo over 6 weeks, indicating a positive impact on secondary outcomes, rather than primary endpoints [72]. In another factorial, blinded, 4-arm randomized clinical trial in Iranian women, both soy flavonoids alone and in combination with vitamin D improve the IBS total score (*p* < 0.05), suggesting a potential synergistic effect; however this was observed in a small, geographically and demographically specific sample [73]. Propolis supplementation, a bee-derived product rich in flavonoids, was shown in a randomized, double-blind clinical trial to reduce IBS-SSS scores and both the severity and frequency of abdominal pain compared with placebo (*p* < 0.05) [74].

### 3.2. Aloe vera

*Aloe vera* (aloe-emodin, aloin, polysaccharides, other anthraquinones/phenolics), as a plant from the Liliaceae family, is widely used as a laxative due to its main active component, barbaloin. Its pathophysiological action begins when intestinal bacteria metabolize barbaloin into aloe-emodie-9-anthrone, which inhibits the Na^+^/K^+^-adenosine triphosphatase in colonic mucosal cells, stimulates mucus secretion, and promotes the release of prostaglandin-like compounds in the colon [75]. These actions increase intestinal motility and paracellular permeability, thereby increasing water content in the intestinal lumen, and *Aloe vera* has therefore been used as a treatment option for IBS-C in adults [76].

Some meta-analyses suggest a potential benefit, although the overall evidence remains inconsistent and not robust. A meta-analysis of 151 patients with IBS showed that *Aloe vera* was effective and safe compared with placebo, improving IBS symptom scores (standardized mean difference, 0.41; 95% CI, 0.07–0.75; *p* = 0.020, with no difference in adverse events [75]. A post hoc analysis of two randomized, double-blind, controlled studies involving 213 patients also showed symptomatic improvement and confirmed that *Aloe vera* (500 mg Aloe extract + 780 mg inulin per day, per os for four weeks) was safe and well tolerated [77]. However, another meta-analysis of 3 randomized clinical trials found no improvement in symptoms in adult IBS patients who received oral *Aloe vera* compared with placebo (*p* = 0.12, *p* = 0.09, and *p* > 0.05, respectively) [78]. Current evidence does not support the routine use of *Aloe vera* in pediatric IBS [75].

### 3.3. Hypericum perforatum (St John’s Wort)

The aerial parts of St John’s Wort (SJW) or *Hypericum perforatum* (hypericin, pseudohypericin, hyperforin, rutin and other flavonoids) are rich in various bioactive phytochemicals including hypericin, quercetin, luteolin and rutin. SJW modulates serotonin levels and reduces psychological stress, which explains its widespread use as an herbal treatment for depression [79]. A controlled clinical trial involving 20 women with IBS reported that SJW extract (300 mg three times daily for 8 weeks) reduced depression and anxiety scores (*p* < 0.01), visceral sensitivity, abdominal pain, bloating, and composite symptom scores [80]. However, in a 12-week randomized double-blind, placebo-controlled trial, SJW (450 mg twice daily per os) showed lower efficacy than placebo in treating IBS symptoms (*p* = 0.03) [81].

### 3.4. Ginger (Zingiber officinale)

Ginger is derived from the rhizome of *Zingiber officinale* (6-gingerol, 6-shogaol, paradols, zingerone), and its primary active compound is 6-gingerol. In a preclinical study using a rat IBS-D model, 6-gingerol significantly reduced colonic inflammation and edema by modulating NF-κB-dependent pro-inflammatory factors, with effects comparable to rifaximin in improving IBS-related outcomes [82]. Another study reported that substances present in the crude ginger extract exhibit both spasmolytic and spasmogenic effects, mediated through inhibition of cholinergic responses and K^+^-induced contractions [83].

Clinical evidence is controversial. In a clinical trial of 60 patients with mild-to-moderate IBS symptoms, a herbal mixture containing *Zingiber officinale*, *Boswellia carterii,* and *Achillea millefolium* significantly decreased abdominal bloating, abdominal pain, anxiety, and depression scores compared to placebo, while improving quality of life in male patients (*p*-0.01) [84]. In contrast, a pilot double blind randomized controlled trial enrolling 45 IBS patients found no statistically significant improvement in IBS symptoms with ginger alone (*p* > 0.05), although reductions in anxiety and depression were observed [85].

## 4. Plant Extracts Without Demonstrated Efficacy in IBS Management

### 4.1. Curcuma xanthorriza

In contrast to *Curcuma longa*, which has shown potential benefits for IBS symptoms, another botanical species of the *Curcuma genus*, *Curcuma xanthorriza* (xanthorrhizol, curcuminoids, germacrone, sesquiterpenes) (Japanese turmeric), has not demonstrated therapeutic benefit over placebo. In a randomized, double-blind, placebo-controlled trial, pain increased in the *Curcuma xanthorriza* group compared with placebo (*p* = 0.81), abdominal distension decreased more in the placebo group than in the treatment group, and the global assessment of IBS symptoms did not differ significantly between groups [86].

### 4.2. Fumaria officinalis

*Fumaria officinalis* (isoquinoline alkaloids, flavonoids, and organic acids) possesses antispasmodic activity in the gut and has therefore been tested in IBS. However, clinical outcomes were not favorable. In a randomized, double blind, placebo-controlled trial, abdominal pain decreased more in the placebo group, abdominal distension decreased in the placebo group but increased in the *Fumaria officinalis* group (*p* = 0.48), and global assessment of IBS symptom changes and IBS-related psychological stress did not differ significantly among the two treatment groups [86].

### 4.3. Ayurvedic Herbal Compound

An Ayurvedic herbal preparation composed of *Murraya koenigii*, *Punica granatum*, and *Curcuma longa* was studied in a randomized, placebo-controlled crossover trial with a randomized sequence of verum and placebo for each patient. The compound showed no greater improvement in IBS-D symptoms than placebo [87].

## 5. Evidence and Guidelines on the Use of Plant Extracts for the Prevention and Management of Irritable Bowel Syndrome

Given the numerous published studies showing that many plant extracts are effective for IBS prevention and management, physicians often face a dilemma regarding which option to choose for a patient’s management. Below are the recommendations from the international guidelines worldwide on the use of herbal extracts for IBS prevention and treatment of IBS, summarized in Table 1.

### 5.1. American Guidelines

The most recent guidelines of the American College of Gastroenterology (ACG) for IBS treatment were published in 2021. The only plant extract recommended was peppermint oil, administered as enteric-coated capsules, not as liquid formulations (e.g., drops). The statement suggests the use of peppermint oil as a first-line therapy for the short-term relief (2–12 weeks) of global IBS symptoms across all IBS subtypes. According to the GRADE (Grading of Recommendations, Assessment, Development and Evaluation) system, the recommendation is conditional and the quality of evidence is low. The ACG guidelines do not provide specific recommendations on peppermint oil dosing regimens or its co-administration with other IBS pharmacotherapies. Other herbal treatments are not recommended; they are only mentioned in some systematic reviews [10].

The Canadian Association of Gastroenterology (CAG) published its IBS guidelines in 2019. The statement on peppermint oil suggests offering it to IBS patients to improve their symptoms, as a supplementary option at any stage of therapy. This is also a conditional recommendation with low-quality evidence. Although some herbal extracts, such as STW-5, have presented positive results, none of the other plant extracts are recommended because there is insufficient evidence to support any specific herbal product as an individual therapy [88].

Regarding Latin America, the 2024 guidelines of the Mexican Association of Gastroenterology state that peppermint oil (first-line choice) and STW-5 may be used to reduce abdominal pain and global IBS symptoms. These treatment options are reported with low-quality evidence, without GRADE ratings. Other herbal extracts are not mentioned [89].

### 5.2. European Guidelines

Similarly, the British Society of Gastroenterology (BSG) in 2021 recommended only peppermint oil as an effective first line treatment for global IBS symptoms and abdominal pain. It is also reported that the gastro-esophageal reflux is a common side effect of peppermint oil. The GRADE assessment was a weak recommendation with very low-quality evidence. BSG does not recommend another plant extract; peppermint oil is the plant-derived intervention considered for IBS treatment [90].

The United European Gastroenterology (UEG) and the European Society for Neurogastroenterology and Motility (ESNM) do not provide guidelines specifically for IBS treatment. However, they published guidelines for functional bowel disorders (FBD) with diarrhea in 2022. In these reports, antispasmodic agents are recommended as first-line treatment for patients with IBS-D, with a weak recommendation and low-quality evidence. Peppermint oil is listed among the antispasmodic agents, but no specific statement or GRADE rating is provided for it. Other plant extracts are not mentioned [91].

The updated 2021 guidelines of the German Society for Gastroenterology, Digestive and Metabolic Disease (DGVS), and the German Society for Neurogastroenterology and Motility (DGNM) recommend peppermint oil as a first-line IBS treatment for abdominal pain and distension, as a low-quality and optional recommendation (these guidelines do not use GRADE ratings). STW-5 and STW-5 II are also reported as potential treatment options with improvements in IBS-SSS and abdominal pain; however, the recommendation remains based on low-quality evidence. Other herbal extracts (e.g., *Aloe vera* and *Curcuma longa*) are reported but not recommended as IBS treatment options [92].

The 2018 guidelines of the Governing Board of the Polish Society of Gastroenterology recommend peppermint oil as a first-line treatment for reducing overall IBS symptoms, with a strong GRADE recommendation and moderate quality of evidence. Regarding STW-5, the available evidence is insufficient to support a recommendation. Other herbal extracts are not mentioned [93].

### 5.3. Asian Guidelines

The Second Asian Consensus guidelines on IBS in 2019 recommend peppermint oil (as an adjunctive treatment) and the Japanese traditional medicine product kampo for IBS treatment, noting that they may be effective. Both GRADE ratings were weak recommendations with low-quality evidence. In addition, TCM reports that herbal and patent prescriptions may be helpful for some IBS patients, but their efficacy should be validated in high-quality randomized clinical trials. Therefore, in general, and without specific TCM recommendations, the statements are more research-oriented than strong clinical recommendations. The GRADE of evidence was moderate. Other herbal extracts are not mentioned [63].

According to the 2020 guidelines of the Japanese Society of Gastroenterology (JSGE), peppermint oil is recommended as an adjunctive treatment for IBS, with a weak GRADE recommendation and high-quality evidence. Furthermore, the JSGE states that some Kampo medicines may be helpful for IBS and recommends their use. The overall GRADE rating is a weak recommendation, supported by low-quality evidence. Other herbal extracts are not mentioned [62].

In 2025, the Korean Society of Neurogastroenterology and Motility reported that antispasmodic therapies can be used to treat IBS abdominal pain as a primary step, and one such antispasmodic is peppermint oil. They do not provide a GRADE rating and do not mention other plant extracts [94].

Finally, in 2023, the Council of Health Insurance (CHI) of Saudi Arabia combined the ACG, BSG, JSGE, and Second Asian Consensus guidelines. It concluded that peppermint oil is effective for IBS symptoms as a supplementary therapy and is recommended. For STW-5, there is insufficient evidence to support its use in the treatment of IBS [95].

### 5.4. Guidelines for Pediatric IBS Prevention and Treatment

The only guidelines for IBS treatment in children and adolescents have recently been published by the European and North American Societies for Pediatric Gastroenterology, Hepatology and Nutrition (ESPGHAN and NASPGHAN). Enteric-coated peppermint oil capsules are suggested as a supplementary treatment option in children with IBS. The GRADE rating is a conditional recommendation with low-certainty evidence. Other herbal extracts mentioned (e.g., SWT-5, *Curcuma*, *Aloe vera*) are not recommended because they have not been adequately studied in children [96].

## 6. Discussion

A wide and steadily growing range of plant extracts has been investigated for IBS prevention and treatment, though only a minority have progressed beyond preclinical evaluation. While several extracts, most notably peppermint oil, Iberogast, *Curcuma longa*, and selected TCM and Japanese Kampo formulations, have shown promising effects in clinical studies, others have demonstrated adverse outcomes or inconsistent findings. The heterogeneity in clinical effectiveness may be partly explained by variations in their underlying mechanisms of action. Plant extracts such as peppermint oil, which influence several key components of IBS pathophysiology, including smooth muscle contractility, visceral hypersensitivity, microbiota composition, and gut–brain axis signaling, tend to demonstrate more consistent therapeutic benefits. Iberogast and certain TCM/Kampo formulations also exert broader effects compared with single-herb formulations, but their evidence base remains geographically limited and insufficient to support widespread guideline recommendation. In contrast, extracts such as *Curcuma longa* predominantly act on the gut–brain axis, which may contribute to their variable clinical results. These mechanistic distinctions highlight that IBS is a multifactorial disorder, and therapies targeting multiple pathophysiological pathways are more likely to achieve meaningful and reproducible clinical improvements. At present, peppermint oil remains the only plant-derived therapy consistently recommended across global guidelines for both adult and pediatric IBS, underscoring its broad mechanistic activity and relatively strong clinical evidence Across current guidelines, its indication is limited to short-term symptomatic relief during acute phases and does not extend to long-term prevention of symptom recurrence.

Safety considerations are also relevant when evaluating multi-component herbal formulations. For example, Iberogast contains ethanol (31% *v*/*v*); however, based on the product information leaflet and the standardized dosing regimen (20 drops three times daily for four weeks), the estimated daily ethanol exposure in adults is approximately 0.72 g. Observational data in adult IBS populations suggest that alcohol-related symptom triggering typically occurs at substantially higher levels of consumption, indicating that the ethanol content of Iberogast at recommended doses is unlikely to represent a clinically meaningful trigger of IBS symptoms [97]. Further well-designed studies are required to clarify the therapeutic value, safety profile, and long-term role of other plant extracts and broaden the evidence-based treatment options available for this complex disease.

This review has several limitations that should be acknowledged. Significant heterogeneity exists among herbal preparations, including variations in plant species, extraction techniques, standardization, and dosing regimens, which limit direct comparisons across studies. The quality of evidence for many extracts remains low due to small sample sizes, short study durations, or methodological problems. Finally, a great proportion of mechanistic evidence is derived from preclinical models that may not fully explain the complexity of IBS in humans. However, this review synthesizes current evidence on plant extracts in IBS, integrating clinical findings, mechanisms, and guideline recommendations. Its strength is based on the comparison of extracts by both efficacy and mechanistic scope, while also identifying regional differences and gaps in the evidence.

## 7. Future Perspectives

Future research should focus on well-designed, multicenter randomized controlled trials using standardized formulas and clinically relevant outcome measures. Given the multifactorial nature of IBS, emphasis should be placed on plant extracts with diverse biological activity, as these agents may offer more consistent and meaningful therapeutic benefits. Mechanistic studies should aim to clarify how plant extracts influence critical IBS pathways, including motility, visceral sensitivity, immune activation, gut microbiota composition, and gut–brain communication, and identify the specific active constituents responsible for these effects.

There is also an urgent need for studies involving diverse populations to explore potential genetic, cultural, and environmental influences on treatment response. Pediatric populations remain particularly understudied, warranting carefully designed trials assessing safety and efficacy. Finally, integrating plant extracts into personalized medicine approaches, potentially guided by biomarkers and microbiome profiles, may help optimize treatment strategies and clarify their role within the broader therapeutic landscape of IBS.

## Figures and Tables

**Table 1:** Plant Extracts in Worldwide Guidelines for the Prevention and Treatment of Irritable Bowel Syndrome.

Guidelines	Peppermint Oil	Iberogast	Kampo	TCM	Other (e.g., *Curcuma, Aloe vera*)	Reference
**ACG** (2021)	First line/GRADE (2–12 weeks) (Conditional recommendation, Low-quality evidence)	Not suggested	Not mentioned	Not mentioned	Not suggested	[10]
**CAG** (2019)	Supplementary/GRADE (Conditional recommendation, Low-quality evidence)	Not suggested	Not mentioned	Not mentioned	Not suggested	[88]
**Mexican Association of Gastroenterology** (2024)	First line/Low-quality evidence	Supplementary/Low-quality evidence	Not mentioned	Not mentioned	Not mentioned	[89]
**BSG** (2021)	First line treatment/GRADE (Weak recommendation, very low-quality evidence)	Not suggested	Not mentioned	Not mentioned	Not suggested	[90]
**UEG/ESNM** for IBS-D (2022)	As an antispasmodic agent (4–12 weeks): First line/GRADE (Weak recommendation, low quality evidence)	Are not mentioned	Not mentioned	Not mentioned	Not mentioned	[91]
**DGNM** (2021)	First line/Low quality, optional recommendation	Low evidence recommendation	Not mentioned	Not mentioned	Not suggested	[92]
**Polish Society of Gastroenterology** (2018)	First line/GRADE (180–225 mg 2 × 1 for 2–12 weeks) (Strong recommendation, Moderate quality evidence)	Not suggested	Not mentioned	Not mentioned	Not mentioned	[93]
**Asian consensus** (2019)	Supplementary/GRADE (Weak recommendation, low quality evidence	Not mentioned	Recommended/GRADE (Weak recommendation, low quality evidence)	Research-based recommendation/GRADE (Moderate quality evidence)	Not mentioned	[63]
**JSGE** (2020)	Supplementary/GRADE (Weak recommendation, High quality evidence)	Not mentioned	Recommended/GRADE (Weak recommendation, low quality evidence)	Not mentioned	Not mentioned	[62]
**Korean Society of Neurogastroenterology** (2025)	As an antispasmodic (0.6 mL/day): Primary therapy	Not mentioned	Not mentioned	Not mentioned	Not mentioned	[94]
**Saudi Arabia-CHI** (2023)	Supplementary	Not suggested	Not mentioned	Not mentioned	Not mentioned	[95]
**ESPGHAN and NASPGHAN** (2025)	Supplementary/GRADE (Conditional recommendation, low quality evidence)	Not suggested	Not mentioned	Not mentioned	Not suggested	[96]

Abbreviations: ACG: American College of Gastroenterology; CAG: Canadian Association of Gastroenterology; BSG: British Society of Gastroenterology; UEG: United European Gastroenterology; ESNM: European Society for Neurogastroenterology and Motility; IBS-D: Irritable Bowel Syndrome with predominant diarrhea; DGNM: German Society for Neurogastroenterology; JSGE: Japanese Society of Gastroenterology; Saudi Arabia-CHI: Council of Health Insurance of Saudi Arabia; ESPGHAN: European Society for Pediatric Gastroenterology Hepatology and Nutrition; NASPGHAN: North American Society Pediatric Gastroenterology Hepatology and Nutrition; GRADE: Grading of Recommendations Assessment development and evaluation.

## Data Availability

No new data were created or analyzed in this study.
